# The impact of body mass index on survival endpoints among patients with metastatic urothelial carcinoma undergoing treatment with immune checkpoint inhibitors: A real‐world multicenter analysis

**DOI:** 10.1002/cam4.7008

**Published:** 2024-02-09

**Authors:** Shih‐Yu Huang, Po‐Jung Su, Chang‐Ting Lin, Ming‐Chun Kuo, Yi‐Hua Chen, Chia‐Che Wu, Hao‐Lun Luo, Chien‐Hsu Chen, Chih‐Chi Chou, Chun‐Chieh Huang, Chung‐Wen Kuo, Yu‐Li Su

**Affiliations:** ^1^ Division of Hematology Oncology, Department of Internal Medicine, Kaohsiung Chang Gung Memorial Hospital and Chang Gung University College of Medicine Kaohsiung Taiwan; ^2^ Division of Hematology Oncology, Chang Gung Memorial Hospital at Linkou and College of Medicine Chang Gung University Tao‐Yuan Taiwan; ^3^ Department of Urology, Kaohsiung Chang Gung Memorial Hospital and Chang Gung University College of Medicine Kaohsiung Taiwan; ^4^ Department of Pathology, Kaohsiung Chang Gung Memorial Hospital and Chang Gung University College of Medicine Kaohsiung Taiwan; ^5^ Department of Radiation Oncology, Kaohsiung Chang Gung Memorial Hospital and Chang Gung University College of Medicine Kaohsiung Taiwan; ^6^ Cancer center, Kaohsiung Chang Gung Memorial Hospital Kaohsiung Taiwan; ^7^ Genomic & Proteomic core lab, Kaohsiung Chang Gung Memorial Hospital Kaohsiung Taiwan

**Keywords:** body mass index, immune checkpoint inhibitor, overall survival, urothelial carcinoma

## Abstract

**Background:**

Studies on the correlation between high body mass index (BMI) and extended survival among patients receiving immune checkpoint inhibitors (ICIs) have been made, although findings have shown variability. Our research explored the phenomenon of the “obesity paradox” in patients with metastatic urothelial carcinoma (mUC) undergoing treatment with ICIs.

**Materials and Methods:**

We conducted a retrospective analysis of patients diagnosed with mUC who received a minimum of one cycle of ICI treatment at two medical centers in Taiwan from September 2015 to January 2023. Features of patients' clinicopathologic factors, including age, sex, primary or metastatic location, treatment line, and BMI were examined. The primary outcome were overall survival (OS) and progression‐free survival (PFS), which were assessed utilizing the Kaplan–Meier method. We employed the Cox‐regression model to adjust for multiple covariates.

**Results:**

A total of 215 patients were included, with 128 (59.5%) being male, and the median age was 70 years. In the obese group (BMI ≥25 kg/m^2^), patients demonstrated significantly better median OS compared to the non‐obese group (BMI <25 kg/m^2^) (21.9 vs. 8.3 months; *p* = 0.021). However, there was no significant difference in median PFS between the high and low BMI groups (4.7 vs. 2.8 months; *p* = 0.16). Post‐hoc subgroup revealed a survival benefit from ICI treatment in male patients within the BMI ≥25 kg/m^2^ group (HR 0.49, 95% CI 0.30–0.81, *p* = 0.005).

**Conclusion:**

Based on real‐world data from the Asia‐Pacific region, there appears to be a correlation between obesity and prolonged OS in patients receiving ICI treatment for mUC.

## 1 INTRODUCTION

Metastatic urothelial carcinoma (mUC) is an aggressive form of cancer that has a high mortality rate. According to the American Cancer Society, in 2021, an estimated 82,290 new cases of bladder cancer were diagnosed in the Unites States, and approximately 16,710 people died from the disease.^1^ The survival rate for patients with mUC remains low, with a five‐year survival rate of around 5%.^2^ Prior to the revolutionary introduction of immune checkpoint inhibitors (ICI) in the treatment landscape, platinum‐based chemotherapy, among other therapies, was the standard first‐line treatment.^3^, ^4^, ^5^ Treatment protocols that were commonly employed, such as GC (gemcitabine/cisplatin) and MVAC (methotrexate, vinblastine, doxorubicin, and cisplatin), demonstrated a significant objective response rate (ORR) and a median overall survival (OS) of around 14 months.^6^, ^7^ Despite first‐line platinum‐based chemotherapy having up to a 40% objective response rate (ORR), tumors can still develop drug resistance and ultimately become uncontrollable, leading to patient death.

Immunotherapy is a type of cancer treatment that harnesses the body's immune system to fight cancer cells. In recent years, immunotherapy has emerged as a promising treatment option for mUC, especially for patients who have not responded to 1st line chemotherapy.^8^, ^9^ Immunotherapy drugs such as ICI have shown encouraging results in clinical trials, with some patients achieving long‐lasting remissions.^10^, ^11^ However, not all patients respond to ICI, and researchers are striving to identify robust biomarkers that can predict response and survival rates to ICI treatment. Previous studies have shown some demographic factors, such as liver metastasis, ECOG performance status and high neutrophil to lymphocyte ratio (NLR) were independent prognostic factors for patients with mUC.^12^, ^13^, ^14^, ^15^ Nevertheless, there are still many unknown factors that have yet to be discovered.

The nutritional status of cancer patients is a crucial prognostic factor, and body mass index (BMI) is a major surrogate for nutritional status in clinical practice.^16^, ^17^, ^18^ Previous studies have shown that high BMI is associated with an increased incidence of cancer in various sites, including the uterus, kidney, gallbladder, thyroid, and leukemia.^19^ However, whether BMI is a prognostic factor in the era of cancer treatment shifting toward immunotherapy remains unknown. In several cancer types, including non‐small lung cancer (NSCLC), head and neck squamous cell carcinoma (HNSCC), and hepatocellular carcinoma (HCC), high BMI has been associated with better overall survival (OS).^20^, ^21^, ^22^ Conversely, obese patients with renal cell carcinoma (RCC) have a significantly worse OS than normal weight patients.^23^ With regard to the relationship between BMI and survival in metastatic urothelial carcinoma (mUC), limited studies have addressed this issue. Ishihara et al. analyzed a total of 104 patients with mUC who underwent ICI therapy and demonstrated that high BMI, defined as a cutoff of 25, did not affect PFS or OS.^24^ However, a similar study revealed opposite results, showing that high BMI is a better prognostic factor in mUC.^25^ As the role of BMI in the prognosis for mUC remains unknown, more clinical studies are warranted to confirm its significance.

In addition, it should be emphasized that the classification standards for BMI in Asian populations differ from those in Western countries.^26^ The ideal BMI cutoff for Asian patients undergoing ICI treatment remains undiscovered. Therefore, we opted to utilize real‐world evidence (RWE) in Asia to explore the link between BMI and the overall survival of patients with mUC, who received ICI as first or second‐line treatment.

## 2 MATERIALS AND METHODS

### 2.1 Patients and treatments

This study was conducted retrospectively and included patients diagnosed with unresectable or metastatic urothelial carcinoma who received monotherapy with ICI at Kaohsiung Chang Gung Memorial Hospital and Linkou Chang Gung Memorial Hospital in Taiwan. All patients received at least one cycle of ICI treatment, including anti‐PD1 (pembrolizumab and nivolumab) or anti‐PDL1 (atezolizumab, durvalumab and avelumab). Various clinical characteristics of the patients, such as sex, age, ECOG performance status, body weight, body height, line of ICI treatment, primary tumor location, metastatic site, PD‐L1 expression by combined positivity score (CPS), and survival, were thoroughly evaluated. BMI was calculated by dividing the weight (in kilograms) by the square of the height (in meters), and patients were classified based on the Asian‐Pacific classification (26). This study was approved by the Institutional Review Board of the Chang Gung Medical Foundation (201901248B0).

### 2.2 Response evaluation and endpoints

All patients underwent regular follow‐up procedures including physical examinations, laboratory test and imaging survey. The computed tomography (CT) or magnetic resonance imaging (MRI) were performed every 2–3 months for tumor response evaluation by using the Response Evaluation Criteria in Solid Tumors (RECIST) criteria version 1.1. The endpoints of study were progression‐free survival (PFS) and overall survival (OS). PFS was determined by calculation of the interval between the start date of ICI and the date of established progression or death. OS was the interval between the start date of ICI therapy and the date of death from any cause or the date the patient was censored.

### 2.3 Statistics

Differences in baseline characteristics and treatment response among the four groups were assessed using Chi‐square and Fisher's exact tests. Survival analysis was performed utilizing the Kaplan–Meier method and assessed through the log‐rank test. Cox proportional hazard models were employed for multivariable and subgroup analysis using the TableSubgroupCox function in Jstable package. Survival curves were generated using the ggsurvplot function of the survminer R package. All tests were two‐tailed, and a *p* value <0.05 was considered statistically significant in all analyses.

## 3 RESULTS

### 3.1 Patient characteristics

From September 2015 through January 2023, 215 patients with mUC were enrolled in this study, including 128 males (59.5%) and 87 females (40.5%), with a median age of 70 years. The median follow‐up time was 31.3 months. The basic characteristics of patients are shown in Table 1. Female was predominant in the underweight group (91.7%) comparing to other 3 groups. Other variables, such as age, ECOG performance status, primary site, metastatic site, treatment line and Bajorin risk showed no significant difference between 4 groups.

**TABLE 1:** Clinicopathologic characteristics of all patients.

	All (*n*, %)	BMI category (kg/m^2^)				*p* Value
		Underweight (<18.5)	Normal (18.5–23)	Overweight (23–25)	Obese (≥25)	
Age (year)						
<65	68 (31.6)	2 (16.7)	34 (37.8)	11 (26.8)	21 (29.2)	0.33
≥65	147 (68.4)	10 (83.3)	56 (62.2)	30 (73.2)	51 (70.8)	
Sex						
Female	87 (40.5)	11 (91.7)	40 (44.4)	13 (31.7)	23 (31.9)	<0.0001
Male	128 (59.5)	1 (8.3)	50 (55.6)	28 (68.3)	49 (68.1)	
ECOG PS						
0–1	178 (82.8)	8 (66.7)	74 (82.2)	38 (92.7)	58 (80.6)	0.15
≥2	37 (17.2)	4 (33.3)	16 (17.8)	3 (7.3)	14 (19.4)	
Primary site						
Bladder	99 (46.0)	5 (41.7)	39 (43.3)	21 (51.2)	34 (47.2)	0.96
Upper tract	114 (53.0)	7 (58.3)	50 (55.6)	20 (48.8)	37 (51.4)	
Multifocal	2 (1.0)	0	1 (1.1)	0	1 (1.4)	
Visceral metastasis						
No	103 (47.9)	8 (52.6)	39 (43.3)	18 (43.9)	38 (52.8)	0.34
Yes	112 (52.1)	4 (47.4)	51 (56.7)	23 (56.1)	34 (47.2)	
Lymph node metastasis						
No	57 (26.5)	2 (16.7)	24 (26.7)	14 (34.1)	17 (23.6)	0.55
Yes	158 (73.5)	10 (83.3)	66 (73.3)	27 (65.9)	55 (76.4)	
Treatment						
1st line	130 (60.5)	10 (83.3)	56 (62.2)	22 (53.7)	42 (58.3)	0.30
≥2nd line	85 (39.5)	2 (16.7)	34 (37.8)	19 (46.3)	30 (41.7)	
Bajorin risk						
0	87 (40.5)	5 (41.7)	35 (38.9)	16 (39.0)	31 (43.1)	0.62
1	107 (49.8)	6 (50.0)	43 (47.8)	24 (58.5)	34 (47.2)	
2	21 (9.8)	1 (8.3)	12 (13.3)	1 (2.4)	7 (9.7)	
PD‐L1 CPS (%)						
<10	84 (39.1)	6 (50.0)	38 (42.2)	17 (41.5)	23 (31.9)	0.49
≥10	58 (27.0)	1 (8.3)	21 (23.3)	11 (26.8)	25 (34.7)	
Missing	73 (34.0)	5 (41.7)	31 (34.4)	13 (31.7)	24 (33.3)	

Abbreviations: BMI, body mass index; COG, Eastern Cooperative Oncology Group; ECPS, combined positive score; PD‐L1, programmed cell death ligand‐1; PS, performance status.

### 3.2 Treatment response

In evaluations based on patients with best response, the ORR of 4 groups were 36.4% (underweight), 30.8% (normal), 27.0% (overweight), and 32.4% (obese), respectively. The disease control rate (DCR) of 4 groups were 54.5% (underweight), 41.0% (normal), 43.2% (overweight), and 57.4% (obese). There were no significant differences between 4 groups regarding to ORR and DCR. All details are shown in Table 2.

**TABLE 2:** Efficacy of immune checkpoint inhibitor stratified by BMI groups.

	Underweight (<18.5 kg/m^2^)	Normal (18.5–23 kg/m^2^)	Overweight (23–25 kg/m^2^)	Obese (≥25 kg/m^2^)	*p* Value
CR	1 (9.1)	9 (11.5)	3 (8.1)	11 (16.2)	0.44
PR	3 (27.3)	15 (19.2)	7 (18.9)	11 (16.2)	
SD	2 (18.2)	8 (10.3)	6 (16.2)	17 (25.0)	
PD	5 (45.5)	46 (59.5)	21 (56.8)	29 (42.6)	
ORR	4 (36.4)	24 (30.8)	10 (27.0)	22 (32.4)	0.92
DCR	6 (54.5)	32 (41.0)	16 (43.2)	39 (57.4)	0.22

Abbreviations: CR, complete response; DCR, disease control rate; ORR, objective response rate; PD, progressive disease; PR, partial response; SD, stable disease.

### 3.3 Survival outcomes

**FIGURE 1:**
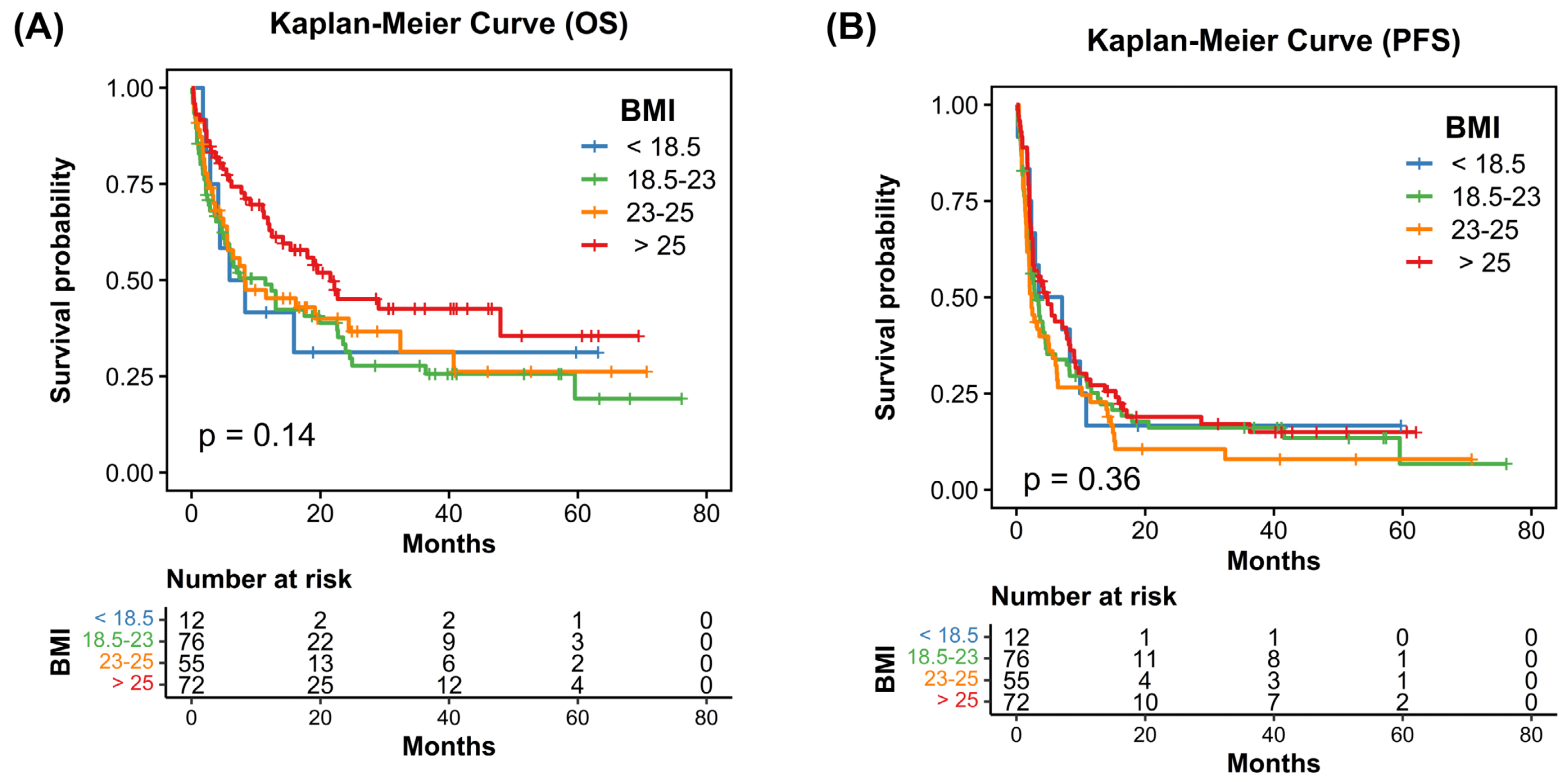
Kaplan–Meier curves of OS (A) and PFS (B) for mUC patients receiving ICIs treatment stratified by BMI groups.

During follow‐up, 128 deaths (8 events in the underweight group, 60 in the normal group, 24 in the overweight group and 36 in the obese group) occurred. The median OS of the 4 groups were 5.9 (underweight), 8.2 (normal), 8.4 (overweight), and 21.9 (obese) months, respectively (log‐rank *p* = 0.14; Figure 1A). Although there was no significant difference between 4 groups analysis, the median OS in the obese group was numerically higher than the other 3 groups. The median PFS of the 4 groups were 3.5 (underweight), 2.8 (normal), 2.5 (overweight), and 4.7 (obese) months, respectively (log‐rank *p* = 0.36; Figure 1B).

**FIGURE 2:**
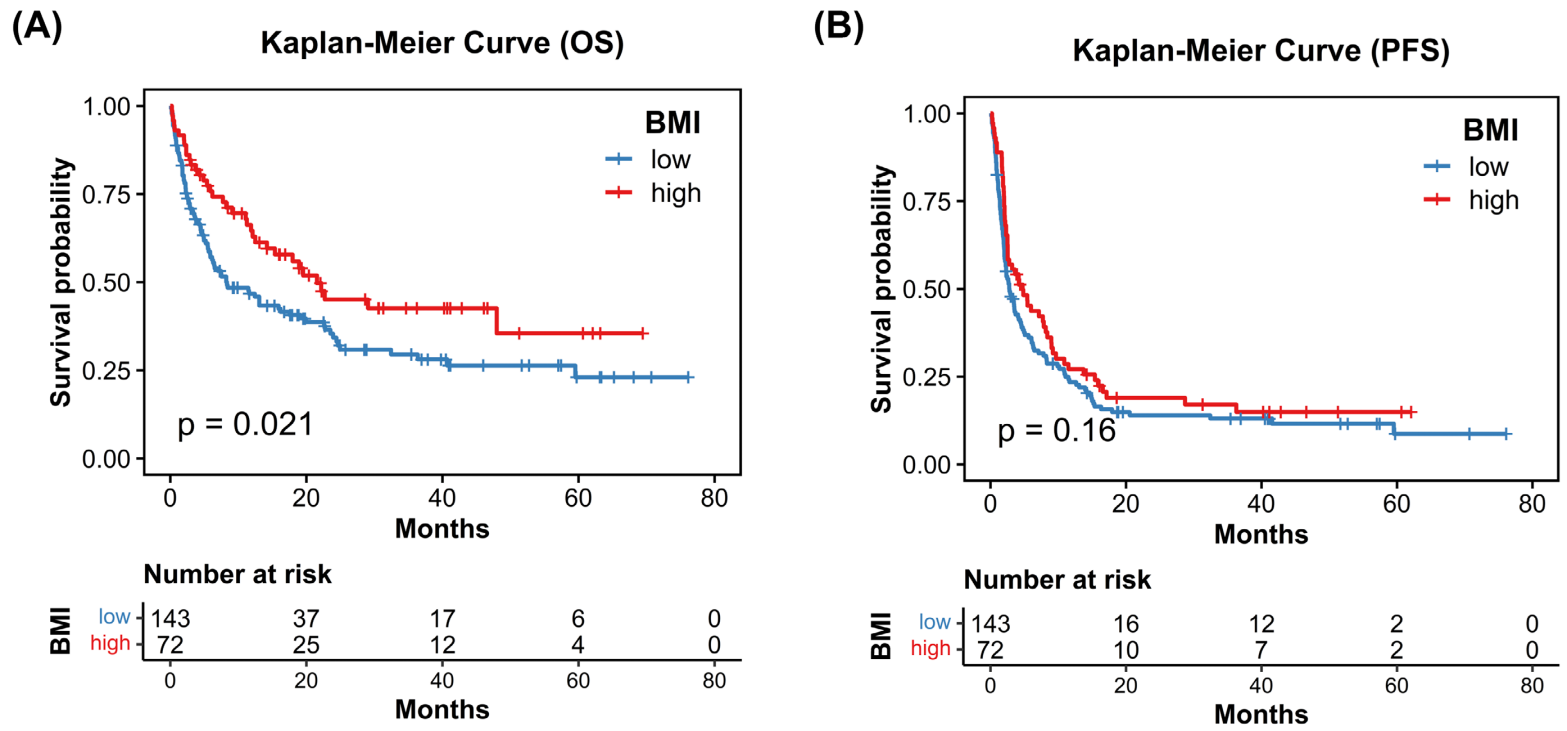
Kaplan–Meier curves of OS (A) and PFS (B) for mUC patients receiving ICIs treatment divided by BMI‐high or BMI‐low groups.

Because the obese group had an excellent good OS, we reclassified our cohorts into BMI‐high (BMI ≥25 kg/m^2^) and BMI‐low (<25 kg/m^2^) to enrich groups number and maximize statistical power. Patients in BMI‐high group had a significant better OS than patients in the BMI‐low group (21.9 vs 8.3 months; *p* = 0.021; Figure 2A). The median PFS showed no significant difference between the BMI‐high and BMI‐low groups (4.7 vs. 2.8 months; *p* = 0.16; Figure 2B).

To understand the other relevant prognostic variables of OS and PFS, we performed univariate analysis and Cox‐regression model for multivariate analysis. In addition to BMI, ECOG PS ≥2 (HR 2.58, 95% CI 1.70–3.90, *p* < 0.0001) and visceral metastasis (HR 2.07, 95% CI 1.45–2.96, *p* < 0.0001) were associated with poor OS (Table 3). In the multivariate analysis, ECOG PS ≥2 (HR 2.94, 95% CI 1.86–4.62, *p* < 0.0001), visceral metastasis (HR 1.93, 95% CI 1.31–2.83, *p* < 0.0001) and BMI ≥25 (HR 0.54, 95% CI 0.35–0.81, *p* = 0.003) were significant associated with overall survival.

**TABLE 3:** Univariate and multivariate analysis of OS.

Characteristics	Median OS	Univariate		Multivariate	
	(month)	HR (95% CI)	*p* Value	HR (95% CI)	*p* Value
Age (year)					
<65	16.2	1	0.76	1	0.65
≥65	12.5	1.06 (0.73–1.53)		1.09 (0.75–1.60)	
Sex					
Female	8.4	1	0.91	1	0.46
Male	17.6	0.98 (0.69–1.39)		1.16 (0.78–1.72)	
ECOG status					
0–1	19.5	1	<0.0001	1	<0.0001
≥2	4.3	2.58 (1.70–3.90)		2.94 (1.86–4.62)	
Origin					
Bladder	18.9	1	0.45	1	0.94
UTUC	8.4	1.15 (0.81–1.63)		1.02 (0.68–1.52)	
LN metastasis					
No	36.4	1	0.02	1	0.07
Yes	11.5	1.64 (1.07–2.52)		1.49 (0.96–2.29)	
Visceral metastasis					
No	29.1	1	<0.0001	1	0.001
Yes	6.2	2.07 (1.45–2.96)		1.93 (1.31–2.83)	
Treatment					
1st line	15.9	1	0.68	1	0.49
≥2nd line	12.1	1.08 (0.76–1.53)		1.14 (0.78–1.66)	
BMI (kg/m^2^)					
<25	8.3	1	0.02	1	0.003
≥25	21.6	0.64 (0.43–0.94)		0.54 (0.35–0.81)	

Abbreviations: BMI, body mass index; CI, confidence interval; ECOG, Eastern Cooperative Oncology Group; HR, hazard ratio; LN, lymph node; OS, overall survival; UTUC, upper tract urothelial carcinoma.

### 3.4 Subgroup analysis of BMI ≥25 kg/m^2^ group

**FIGURE 3:**
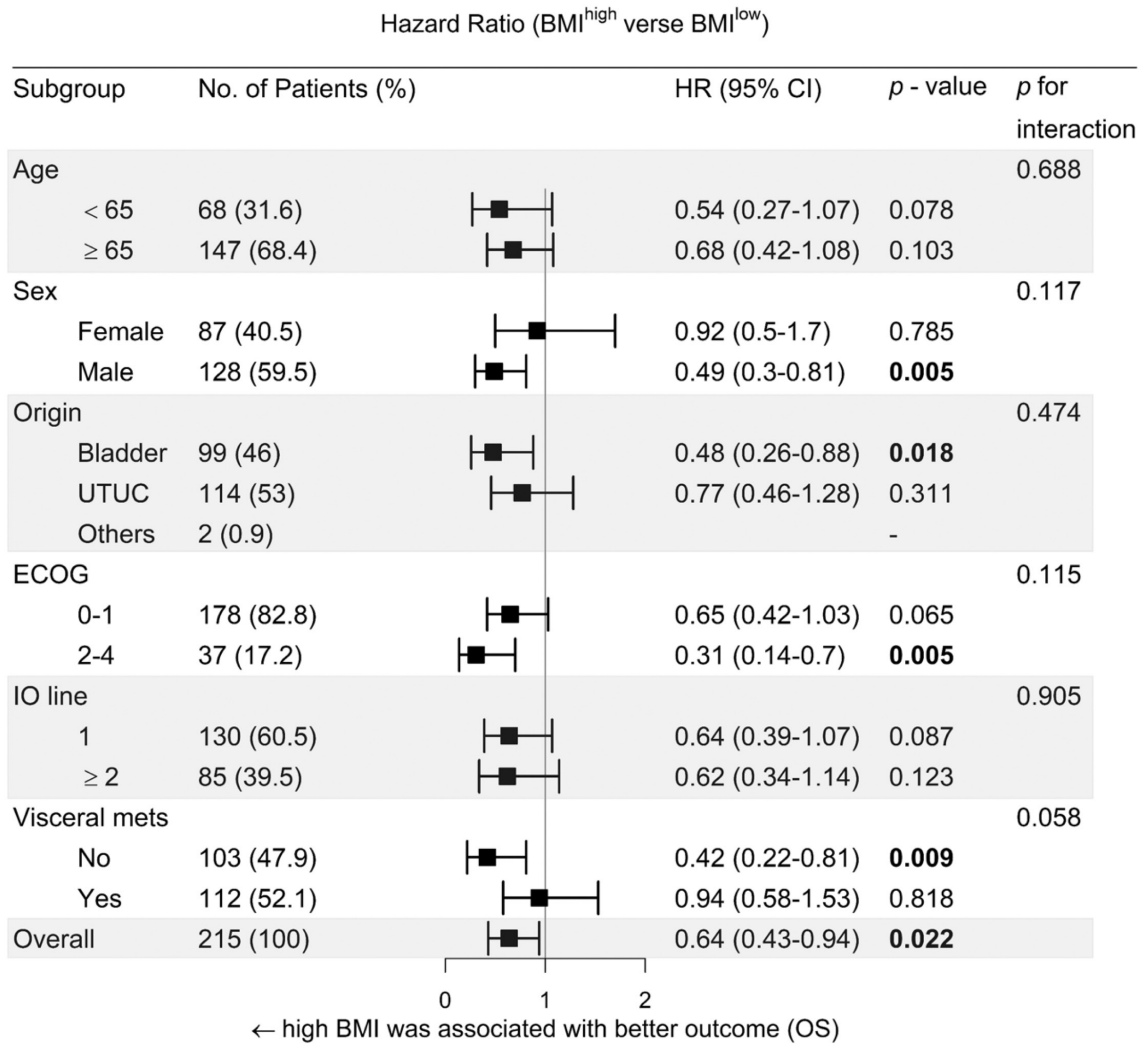
Forest plots of hazard ratios (HRs) for patients with high BMI versus low BMI by clinicopathologic factors.

**FIGURE 4:**
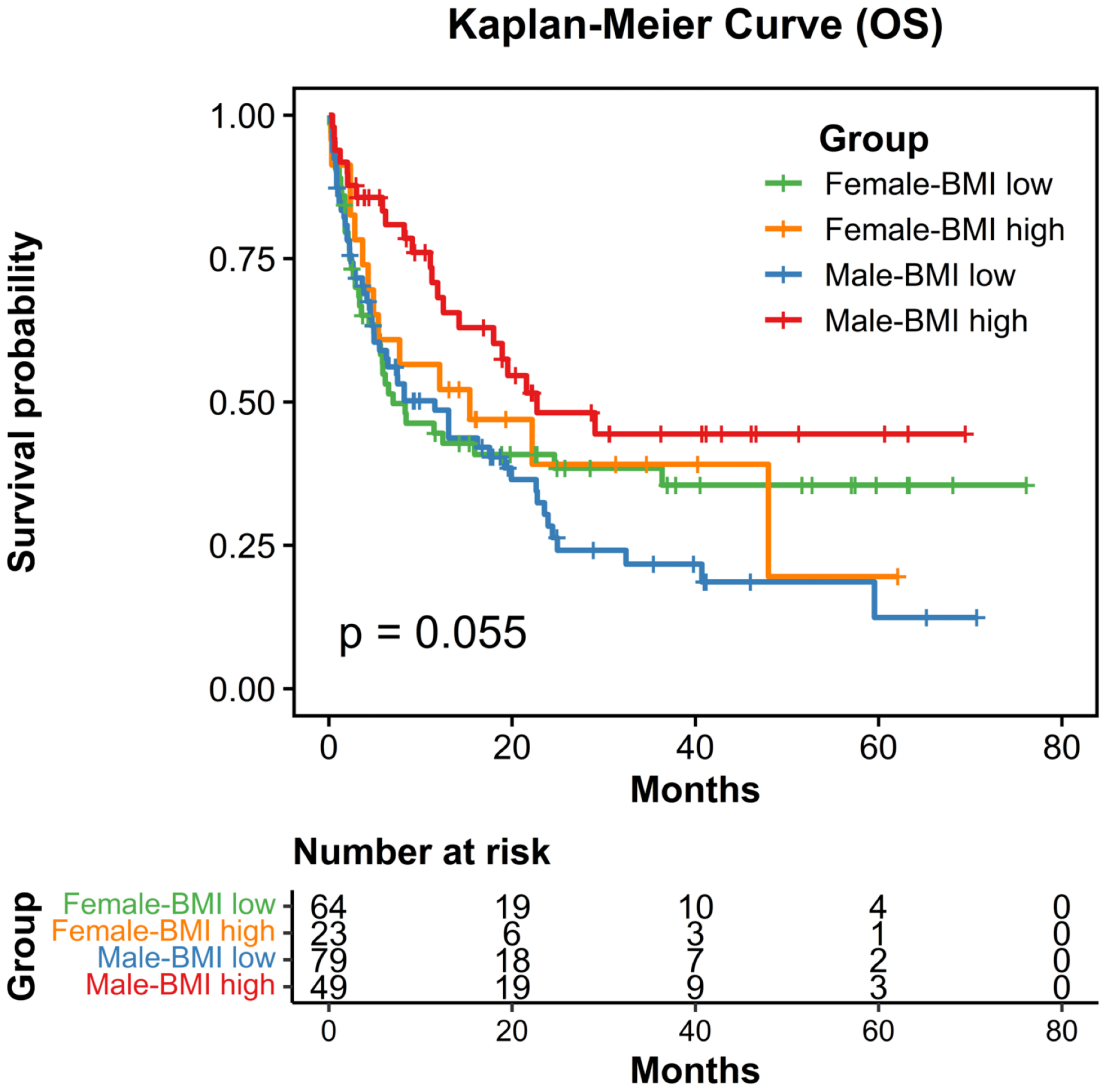
Kaplan–Meier curves of OS for mUC patients receiving ICIs treatment divided by BMI (high or low) and sex (male or female).

To gain a better understanding of which subgroups within the BMI ≥25 kg/m^2^ group may benefit from ICI treatment, we conducted a post‐hoc analysis to examine the subgroups. Patients with some specific variables, such as male (HR 0.49, 95% CI 0.30–0.81, *p* = 0.005), bladder origin (HR 0.48, 95% CI 0.26–0.88, *p* = 0.018), ECOG ≥2 (HR 0.31, 95% CI 0.14–0.70, *p* = 0.005) and no visceral metastasis (HR 0.42, 95% CI 0.22–0.81, *p* = 0.009) may gain a survival benefit trend toward using ICI in univariate analysis (Figure 3). Additionally, we observed a survival trend within the obesity group stratified by sex (*p* = 0.055; Figure 4). Nevertheless, we cannot assert the significance of survival benefits for male patients receiving ICI, as the *p* value for interaction did not reach statistical significance (*p*_interaction_ = 0.117).

## 4 DISCUSSION

Despite the accumulating evidence suggesting a favorable association between high BMI and clinical outcomes in individuals receiving ICI therapy, the current literature remains limited in terms of its ability to substantiate this observation specifically in patients with mUC. Our study demonstrated that BMI ≥25 is an independent factor to predict OS in mUC patients on ICI treatment, but not a determining factor for ORR and PFS. Furthermore, we found that male patients and those without visceral metastasis experienced greater benefits from ICI treatment within the BMI ≥25 group. To our best knowledge, this is the largest study to investigate survival impact of BMI in mUC.

There is an undeniable connection between obesity and a heightened susceptibility to specific types of cancers, such as esophageal adenocarcinoma and colon cancer in man, as well as endometrial cancer in women.^27^, ^28^ Many studies have also confirmed that patients with high BMI exhibited notably elevated rates of recurrence and diminished cancer‐specific survival (CSS) in cases of breast, prostate and colorectal cancer.^29^, ^30^, ^31^ However, there is also an increasing amount of evidence indicating that in certain specific cancers, patients with higher BMI exhibit better OS compared to patients with normal BMI.^32^, ^33^, ^34^, ^35^ The phenomenon referred to as the “obesity paradox” presents a multifaceted and intriguing puzzle in the realm of cancer studies.^36^ The paradoxical nature of this phenomenon might be explained by the notion that tumors in obese patients could exhibit less aggressive characteristics, especially in metastatic status. In a comprehensive meta‐analysis conducted by Modi et al., it was observed that in the case of early‐stage HER2‐positive breast cancer, obese patients exhibited significantly worse overall survival. Conversely, among patients with advanced or metastatic HER2‐positive breast cancer, obese individuals demonstrated a notably improved OS. This finding imply that obesity might exert an influence on host immunity, the tumor microenvironment and the mechanism regulating disease progression.^37^ Furthermore, it is essential to emphasize that obesity exerts a detrimental influence on the response to neoadjuvant chemotherapy in breast cancer patients. This observation suggests that the survival advantages associated with obesity may not arise from an enhancement in treatment efficacy but rather from the modulation of tumor behavior and host‐related factors.^38^ In our analysis, we observed a similarity in ORR between the underweight and obese groups, yet a substantial difference in OS emerged. Investigating the reasons behind the lack of divergence in ORR despite a significant difference in OS remains a subject for further exploration. Nevertheless, depending on ORR as a predictor for OS in the era of ICI treatment lacks substantial evidence. Numerous comprehensive meta‐analysis studies have consistently demonstrated a weak correlation between ORR and OS.^39^, ^40^, ^41^ This intriguing and paradoxical finding challenges established assumptions and urges further investigation into the potential mechanisms driving this phenomenon, with particular attention directed toward immunological factors.

As cancer treatment advances into the era of ICI therapy, it is essential to reassess the truth of the “obesity paradox” in patients with metastatic cancer. In a substantial cohort study comparing the effectiveness and survival outcomes of first‐line pembrolizumab versus chemotherapy, Cortellini et al. demonstrated that a higher baseline BMI was correlated with increasing ORR, PFS and OS in NSCLC. Conversely, in the case of obese patients receiving chemotherapy, no analogous survival trend was observed.^20^ In the context of mUC, there was a paucity of data investigating the association between obesity and survival. Tomisaki and colleagues conducted an analysis involving 84 patients with mUC undergoing pembrolizumab treatment, revealing a noteworthy extension in PFS within the overweight group compared to the non‐overweight group.^25^ Despite observing a numerical increase in ORR and OS within the overweight group, statistical significance was not achieved. Another comparable study by Ishihara demonstrated that neither PFS nor OS showed significant differences between the high BMI and low BMI groups in the mUC cohort.^24^ Our study represents the most extensive cohort validating the survival impact of BMI in the era of ICI treatment. It is important to highlight that our study cohort comprised nearly 60% of mUC patients receiving first‐line ICI treatment. This stands in contrast to Ishihara's cohort, which exclusively encompassed patients receiving second or third‐line ICI treatments.

Despite extensive research into the precise mechanisms and pathophysiology of the obesity paradox, a definitive conclusion has yet to be reached. Excessive adiposity leads to a chronic state of inflammation, which has been linked to an increased risk of cancer development and progression.^42^ Recent studies have intriguingly revealed that adipose tissue can exhibit dual effects in tumor environments, promoting both pro‐tumor and anti‐tumor effects by modulating distinct types of innate immune cells. Additionally, it has been found to induce dysfunction in PD‐1‐related T cells. Wang and colleagues showcased that in diet‐induced obese (DIO) mice, there was a notable increase in exhausted T cells via leptin pathway, ultimately promoting tumor progression and metastasis.^43^ Intriguingly, the administration of anti‐PD1 in DIO mice led to remarkable tumor regression when compared to B16 mice. Moreover, upon analyzing the TCGA database, which included 250 cancer patients treated with PD‐1/PD‐L1 inhibitors, the tumor microenvironment phenotype demonstrated a notable increase in PD‐1, TIM‐3, LAG3, TIGIT, T‐bet, and EOMES in tumors of obese patients. These studies lay the fundamental groundwork for understanding the underlying mechanism of the obesity paradox in patients with metastatic cancer.

Our study is unquestionably affected by several limitations. These encompass a retrospective design that entails inherent risks of selection bias and data collection biases. The absence of a centralized imaging review for response assessment and population heterogeneity are additional constraints. Furthermore, the lack of comprehensive data on immunotherapy related adverse effect (irAE) and the absence of a control group who did not receive ICIs further reduce the robustness of our analysis. However, a significant advantage of our study is the evaluation of BMI's prognostic potential based on real‐world data, and notably, our study enrolled the highest numbers of mUC patients compared to previous research. Last but certainly not least, it is crucial to recognize the complexity of body composition, emphasizing that BMI may not be a perfect measure of adiposity. Future research should consider utilizing more comprehensive measures of adiposity and prospective study designs to further validate and refine the observed associations.

## 5 CONCLUSION

Our study illustrated that patients with mUC and obesity (BMI ≥25 kg/m^2^) experience significant overall survival benefits when undergoing ICI therapy.

## AUTHOR CONTRIBUTIONS

**Shih‐Yu Huang:** Conceptualization (supporting); writing – original draft (equal). **Po‐Jung Su:** Formal analysis (lead). **Chang‐Ting Lin:** Formal analysis (supporting); software (supporting). **Ming‐Chun Kuo:** Methodology (lead). **Yi‐Hua Chen:** Software (lead); visualization (lead). **Chia‐Che Wu:** Data curation (equal). **Hao‐Lun Luo:** Data curation (equal). **Chien‐Hsu Chen:** Data curation (equal). **Chih‐Chi Chou:** Data curation (equal). **Chun‐Chieh Huang:** Data curation (equal). **Chung‐Wen Kuo:** Writing – original draft (supporting). **Yu‐Li Su:** Conceptualization (lead); writing – original draft (equal); writing – review and editing (lead).

## FUNDING INFORMATION

The study was supported partly by a grant from Chang Gung Memorial Hospital, Kaohsiung, Taiwan (CMRPG8M0651, CMRPG8K1661, CPRPG8J0012).

## CONFLICT OF INTEREST STATEMENT

The authors have no conflict of interest.

## ETHICS STATEMENT

Approval of the research protocol by an Institutional Reviewer Board: This study was approved by the Institutional Review Board of the Chang Gung Medical Foundation (201901248B0).

## Data Availability

Access to the data supporting the study's conclusions can be requested from the corresponding author.
